# A retrospective analysis of antimicrobial prescriptions in dentistry: Understanding the patterns

**DOI:** 10.4317/jced.64329

**Published:** 2026-09-15

**Authors:** Joana Magalhães Silva, Maria Gonçalves, Maria do Pranto Valente Braz, Cristina Maria Leal Moreira Coelho

**Affiliations:** 1 CESPU University, 4585-116 Gandra PRD, Portugal; 2 UNIPRO - Oral Pathology and Rehabilitation Research Unit, CESPU’S University, 4585-116 Gandra, Portugal; 3 Associate Laboratory i4HB - Institute for Health and Bioeconomy, CESPU University, 4585-116 Gandra, Portugal; 4 UCIBIO - Research Unit on Applied Molecular Biosciences, Toxicologic Pathology Research Laboratory, CESPU University, 4585-116 Gandra, Portugal; 5 UNIPRO - Oral Pathology and Rehabilitation Research Unit, CESPU’S University, 4585-116 Gandra, Portugal

**Keywords:** Antibacterial agents, dentistry, prescriptions, drug resistance, microbial.

## Abstract

**Background:**

The widespread use of antibiotics, particularly amoxicillin and amoxicillin-clavulanic acid, has improved the management of bacterial infections. However, their inappropriate use contributes to antimicrobial resistance, a major public health concern. Dentists account for approximately 10% of antibiotic prescriptions worldwide, often based on empirical decisions. This study aimed to analyse antibiotic prescribing patterns and assess adherence to clinical guidelines in a University Dental Clinic.

**Materials and Methods:**

A retrospective observational study was conducted using clinical records from patients treated between January 2021 and December 2025. Data included sociodemographic variables, prescription characteristics, and guideline-based appropriateness. Statistical analysis was performed using SPSS version 31.0, with significance set at p<0.05.

**Results:**

A total of 1770 patient records were included, of which 14.1% involved a prescription. Amoxicillin-clavulanic acid was the most frequently prescribed antibiotic (55.0%). Only 20.1% of prescriptions were appropriate, while 36.9% were inappropriate and 43.0% could not be assessed because of incomplete documentation. Antibiotic prescribing was significantly associated with dental service (p<0.001).

**Conclusions:**

Antibiotic prescribing practices showed substantial deviations from guideline recommendations, particularly due to frequent broad-spectrum antibiotic use and incomplete clinical documentation. These findings underscore the need to strengthen antimicrobial stewardship and improve record quality in dental settings.

## Introduction

The introduction of antibiotics, drugs used to treat and prevent bacterial infections by inhibiting or eliminating bacterial growth [1], in the 20th century revolutionized the management of infectious diseases, which were previously a leading cause of mortality. However, their widespread and often inappropriate use has contributed to the emergence of antimicrobial resistance (AMR), now recognized as a major global public health threat [2,3].

Antibiotics comprise several pharmacological classes with distinct mechanisms of action, including nitroimidazoles, macrolides, lincosamides, fluoroquinolones, tetracyclines, and beta-lactams. Among these, amoxicillin and amoxicillin-clavulanic acid remain the most frequently prescribed agents in dental practice [4]. Although AMR is a natural evolutionary process, its accelerated spread is strongly associated with the misuse and overuse of antibiotics [5]. In Europe and the United States, AMR is responsible for approximately 37,000 and 50,000 deaths annually, respectively [6].

Dentists play a significant yet often underrecognized role in antibiotic stewardship, accounting for approximately 10% of global antibiotic prescriptions. Evidence suggests that many prescriptions are empirical, with up to 66% lacking clear clinical indication [7-9]. This contributes to the frequent use of broad-spectrum antibiotics, despite recommendations favouring targeted, narrow-spectrum therapy whenever possible [4]. In addition, non-clinical factors, such as perceived patient expectations, may influence prescribing behaviour, further increasing the risk of inappropriate use [10].

To address these concerns, clinical guidelines have been developed to support evidence-based decision-making. In dentistry, antibiotics should be considered adjunctive to operative or mechanical interventions, which remain the primary approach for managing most oral infections [11]. Therefore, evaluating real-world prescribing practices is essential to determine whether current clinical decisions align with established recommendations.

Most existing studies in Dentistry rely on self-reported data obtained through questionnaires, which may be subject to reporting bias and inaccuracies. Retrospective analyses of clinical records offer a more objective assessment of prescribing behaviour.

Although previous studies have examined antibiotic prescribing patterns among Portuguese oral healthcare professionals, mainly through surveys, to our knowledge, this is the first retrospective study to exclusively analyse the clinical practice of undergraduate dental students in a Portuguese University Clinic, while simultaneously assessing both guideline adherence and the quality of clinical documentation.

The aim of this study was to analyse antibiotic prescribing patterns, identify potential inappropriate use, and assess adherence to clinical guidelines through a retrospective evaluation of patient records from a University Dental Clinic in Gandra, Porto, Portugal.

## Materials and Methods

*Design*

A retrospective observational analytical study was conducted at the University Dental School Clinic, based on electronic clinical records from patients treated between January 2021 and December 2025, across multiple fields of Dentistry. Data were progressively collected throughout the study period, which ended in December 2025, and the final dataset was reviewed in January 2026. The study protocol was approved by the Clinical Director, as well as by the Ethics Committee of the University Institute of Health Sciences - CESPU (File no. 33/CE-IUCS/2024). The study was carried out in accordance with institutional ethical regulations and the Strengthening the reporting of observational studies in epidemiology (STROBE) checklist for observational studies.

*Data collection*

Clinical information was retrieved from the University clinic's electronic management system. An initial extraction was performed to obtain patient record numbers and the date of the last consultation, which allowed cases to be stratified by year. These data were then used to access individual patient records directly, avoiding the need to review appointments case by case in the scheduling system.

For each eligible consultation, the clinical history was reviewed to identify documented drug allergies, followed by an examination of clinical notes and prescription records. The following variables were collected: Date of consultation, gender, age, prescribed antibiotic, generic or brand-name presentation, dosage, duration of treatment, indication for prescription, reason for consultation, occurrence of adverse drug reactions, dental service, need for a new prescription, number of prescriptions issued, and guideline appropriateness.

A descriptive year-by-year assessment of the distribution of records was performed. However, formal temporal trend analysis of antibiotic prescribing rates was not conducted because, for patients without antibiotic prescription, the date used for coding corresponded to the last clinical visit rather than to the date of a specific antibiotic prescribing decision. Therefore, year-by-year comparisons could introduce temporal misclassification and were considered unsuitable for trend interpretation.

To ensure confidentiality, patients were identified exclusively by case number. Data extraction was performed by a single calibrated investigator to promote consistency across records. The appropriateness classification of all prescriptions was independently reviewed by a second investigator using the same predefined criteria. Disagreements were resolved by discussion until consensus was reached. To assess reproducibility, a random subsample of records was independently classified by a second reviewer, and inter-rater agreement for appropriateness classification was estimated using Cohen’s kappa (k=0.83).

*Sampling strategy*

A non-probabilistic consecutive sampling approach was used. All records that met the inclusion criteria were screened consecutively to minimize selection bias. Patients of all age groups were eligible, regardless of whether an antibiotic had been prescribed. Only consultations performed by undergraduate dental students were included. Consultations performed by faculty members or postgraduate dental students were excluded to focus on undergraduate practice, reducing variability related to undergraduate training obtained at different institutions. 

*Assessment of appropriateness*

Appropriateness of antibiotic prescriptions was assessed according to current clinical guidelines from the European Federation of Periodontology (EFP), European Society of Cardiology (ESC), European Society of Endodontology (ESE), and American Academy of Pediatric Dentistry (AAPD) depending on the clinical indication, considering antibiotic indication, selection, dosage, and duration of therapy. Prescriptions were classified as appropriate, inappropriate, or indeterminate when essential information was missing from the record and did not allow a reliable assessment (Supplementary Table 1: https://www.medicinaoral.com/medoralfree01/64329/Suppl1.pdf).

*Statistical analysis*

Statistical analysis was performed using IBM® SPSS® Statistics software (Statistical Program for Social Sciences), version 31.0 for Windows (IBM, New York, NY, USA). Descriptive statistics were expressed as mean and standard deviation for quantitative variables, and as frequencies and percentages for qualitative variables. The association between antibiotic use and field of Dentistry was assessed using the Chi-square test. Effect sizes were measured using Phi (Φ). The level of significance was set at *p*<0.05.

## Results

*Characterization of sample*

The initial sample included 2000 patient records, of which 230 were excluded from the final analysis. Among the excluded, 83 were conducted by faculty members and 74 by postgraduate dental students. In 3 cases, the recorded information was incomprehensible. Additionally, 50 records contained no available data in the system, 17 lacked age information, and 3 lacked gender information.

After applying the exclusion criteria, the final sample comprised 1770 patient records, with ages ranging from 1 to 94 years, with a mean age of 44.20 ± 19.80 years. Of these patients, 55.8% were female and 44.2% male. Antibiotics were prescribed in 14.1% of cases (n=249).

The distribution of records by year was 37 in 2021, 58 in 2022, 95 in 2023, 683 in 2024 and 897 in 2025.

*Type of antibiotic prescribed*

Among the 249 patients who received antibiotic therapy, the most frequently prescribed drug was Amoxicillin combined with Clavulanic acid (55.0%), followed by Amoxicillin alone (17.3%). In 16.5% of cases, antibiotic therapy was reported without specification of the drug. Other antibiotics, including Clarithromycin and Azithromycin were prescribed less frequently (Table 1).

*Antibiotic dosages*

The most used dosage was 875 mg + 125 mg (41.0%) of Amoxicillin with Clavulanic acid. However, dosage information was not recorded in 36.1% of prescriptions, representing a substantial proportion of incomplete records (Table 2). This limitation reduced the ability to verify whether prescribed regimens were consistent with guideline recommendations.

*Treatment duration*

Most antibiotic treatments had a duration of 8 days, representing 53.8% of cases. Shorter treatment durations were considerably less frequent. A small number of cases involved prophylactic regimens, including one prescription administered 1 hour before the appointment, after the appointment and the following day (0.4%), and two prescriptions administered 1 hour before the appointment (0.8%). In 31.7% of cases, the duration of treatment was not reported (Table 3).

*Association between antibiotic use and dental service*

Analysis of the association between dental service and antibiotic prescription revealed statistically significant differences (χ²=40.43; *p*<0.001) (Table 4). General dentistry accounted for the highest proportion of antibiotic prescriptions (56.6%), followed by Oral Surgery and Periodontology (21.3%). In contrast, Paediatric Dentistry (3.2%) and Oral Rehabilitation (2.8%) presented considerably lower prescription rates. The effect size was small (Φ=0.151), indicating that service type was associated with prescribing but was not the only influencing factor.

*Appropriateness according to guidelines*

Regarding adherence to reference guidelines, only 20.1% (n=50) of prescriptions were considered appropriate, whereas 36.9% (n=92) were classified as inappropriate. In addition, 43.0% (n=107) could not be evaluated because of insufficient information in the clinical records. Among the 142 that could be reliably assessed, 92 (64.8%) were classified as inappropriate, while 50 (35.2%) were considered appropriate. Among the inappropriate prescriptions, the most frequent deviation was antibiotic use in clinical situations where it was not recommended by the reference guidelines (n=56), followed by inappropriate drug selection (n=23), inappropriate duration (n=11), and inappropriate dosage (n=2).

## Discussion

The present study found that 14.1% of consultations resulted in an antibiotic prescription, with amoxicillin-clavulanic acid being the most frequently prescribed agent (55.0%). This pattern is consistent with findings by Albuquerque *et al*., who reported a similar preference for broad-spectrum antibiotics among Portuguese oral health professionals, despite guidelines recommending narrower spectrum as first-line therapy [12]. These results align with previous studies indicating the continued reliance on broad-spectrum antibiotics in dental practice [13].

Although the use of amoxicillin-clavulanic acid may be justified in specific clinical situations, such as suspected beta-lactamase-producing infections, failure of first-line therapy, or systemic involvement, its routine use in the absence of such indications is not supported. In cases without systemic signs, local mechanical intervention remains the gold standard, and antibiotics should be considered adjunctive therapy [14,15]. However, in the present study, documentation did not always allow these clinical considerations to be verified.

A major finding of this study was the inconsistency and incompleteness of clinical documentation. A substantial proportion of prescriptions lacked essential information, including dosage (36.1%) and treatment duration (31.7%), which limited the ability to fully assess prescribing quality. In addition, 43.0% of prescriptions could not be evaluated for guideline compliance due to insufficient data. This limitation has been reported in other studies and remains a major barrier to accurately assessing prescribing practices [16-19]. Accurate documentation is essential not only for evaluating adherence to guidelines but also for ensuring continuity of care and supporting the identification of adverse drug reactions.

Despite these limitations, no adverse drug reactions were reported among the 249 prescriptions, which is consistent with short-term antibiotic use in supervised clinical settings. However, incomplete documentation may also hinder the detection of rare adverse events.

The observed association between dental services and antibiotic prescribing suggests that clinical context influences prescribing decisions. Higher prescription rates in General Dentistry (56.6%) likely reflect its triage function, while Oral Surgery and Periodontology (21.3%) involve more invasive procedures that may require antibiotic coverage in selected cases. This association was statistically significant, although the small effect size indicates that additional factors also contribute to prescribing variability.

Interpretation of guideline adherence requires caution. Although a relatively low proportion of prescriptions were classified as appropriate [15-20], this may partly reflect the empirical nature of antibiotic prescribing in dentistry [21]. In clinical practice, treatment decisions are often made without bacteriological confirmation, which may lead clinicians to adopt more cautious approaches, including the use of broad-spectrum antibiotics. Furthermore, antibiotics may be prescribed in situations where local mechanical treatment is not immediately feasible, such as in cases of severe inflammation that compromise the effectiveness of local anaesthesia [22]. Deviations from recommended antibiotic regimens, including variations in dose and duration, were observed and may contribute to AMR [20,23].

Similarly, for conditions such as dental abscesses, guidelines recommend drainage whenever possible [24]. However, the absence of documentation about whether drainage was feasible may have led to some prescriptions being classified as inappropriate. When excluding non-assessable cases (43.0%), the proportion of appropriate prescriptions increased from 20.1% to 35.2%, suggesting a more nuanced interpretation of prescribing practices. Some of those prescriptions may in fact have been clinically justified but could not be evaluated from the available records.

Our findings are consistent with studies from other countries reporting substantial levels of inappropriate antibiotic prescribing in dentistry [25-27]. While Durkin *et al*. (2018) reported lower rates of inappropriate prescribing (14%) [26], other studies have documented considerably higher proportions, ranging from 41% to 92% [28,29]. Additionally, Gelda *et al*. (2026) reported a higher overall prescribing rate (38.7%) compared to the present study [29]. These variations may reflect differences in study design, clinical settings, and definitions of appropriateness. The present study contributes to this literature by providing data from a University Dental Clinic in Portugal and by combining prescribing patterns with an assessment of record completeness.

A key factor underlying these prescribing patterns is the limited use of bacteriological testing in dental practice [30]. Unlike other medical disciplines, dental prescribing is largely empirical, which may contribute to the preference for broad-spectrum antibiotics as a precautionary measure [21].

This study has several limitations. The high proportion of incomplete records restricted the assessment of guideline adherence, and the single-centre design limits the generalizability of the findings. In addition, the retrospective design prevented verification of undocumented clinical reasoning, so some prescriptions classified as inappropriate may have been clinically justified but insufficiently recorded.

## Conclusions

Overall, these findings underscore the need to strengthen antimicrobial stewardship strategies in dental practice. Improving the completeness and accuracy of clinical records is essential to support appropriate prescribing and ensure continuity of care. Given the contribution of Dentistry to global antibiotic use, targeted interventions are necessary to reduce inappropriate prescribing and mitigate the progression of AMR.

## Supplementary Material

Supplementary 1

## Figures and Tables

**Table 1:** Table 1: Type of antibiotic prescribed (n=249).

**Type of Antibiotic**	**n**	**%**
Amoxicillin + Clavulanic Acid	137	55.0
Amoxicillin	43	17.3
Received antibiotic therapy without specification of the drug	41	16.5
Clarithromycin	16	6.4
Azithromycin	6	2.4
Amoxicillin + Metronidazole	2	0.8
Clindamycin	2	0.8
Amoxicillin + Clavulanic Acid + Metronidazole	1	0.4
Metronidazole	1	0.4
Total	249	100.0

**Table 2:** Table 2: Dosage of antibiotics prescribed.

**Antibiotic Dosage**	**Respective Antibiotic**	**n**	**%**
875 mg + 125 mg	Amoxicillin with Clavulanic acid	102	41.0
1 g	Amoxicillin	23	9.2
500 mg	Clarithromycin, Azithromycin	21	8.4
2 g	Amoxicillin	2	0.8
12.5 ml per dose	Amoxicillin with Clavulanic acid	1	0.4
25 mg/ml - 1 spoon according to body weight	Clarithromycin	1	0.4
125 mg + 31.25 mg	Amoxicillin with Clavulanic acid	1	0.4
150 mg	Clindamycin	1	0.4
250 mg	Metronidazole	1	0.4
1 g + 250 mg	Amoxicillin + Metronidazole	1	0.4
400mg + 57 mg	Amoxicillin with Clavulanic acid	1	0.4
500 mg + 125 mg	Amoxicillin with Clavulanic acid	1	0.4
500 mg + 250 mg	Amoxicillin + Metronidazole	1	0.4
600 mg + 42.9 mg	Amoxicillin with Clavulanic acid	2	0.8
Not reported		90	36.1
Total		249	100.0

**Table 3:** Table 3: Duration of antibiotic therapy.

**Duration of antibiotic therapy**	**Respective Antibiotic**	**n**	**%**
10 days	Clarithromycin	3	1.2
8 days	Amoxicillin with Clavulanic acid or Amoxicillin isolated	134	53.8
7 days	Amoxicillin with Clavulanic acid	5	2.0
6 days	Clarithromycin	10	4.0
5 days	Amoxicillin	5	2.0
4 days	Unsubstantiated therapeutic choice	2	0.8
3 days	Azithromycin	8	3.2
1 h before the appointment, after the appointment and the following day	Amoxicillin	1	0.4
1 h before the appointment	Amoxicillin	2	0.8
Not reported		79	31.7
Total		249	100.0

The 7-day duration presented is consistent with the therapeutic regimen of amoxicillin with clavulanic acid, corresponding to the 8-day treatment course (875 mg + 125 mg). The 7 days represent a typographical error in the clinic's program. The regimen of 1-hour before the appointment, after the appointment and the following day is not consistent with current guidelines based on ESC 2023.

**Table 4:** Table 4: Association between antibiotic prescription and dental service.

	**Antibiotic**					
**Service**	**No**	**Yes**	**Total**	**χ** ^2^	*p*	**Φ**
Oral Surgery and Periodontology	243 (16.0)	53 (21.3)	296			
Restorative Dentistry	173 (11.4)	40 (16.1)	213			
General Dentistry	751 (49.4)	141 (56.6)	892			
Paediatric Dentistry	158 (10.4)	8 (3.2)	166	40.43	<0.001	0.151
Oral Rehabilitation	196 (12.9)	7 (2.8)	203			
Total	1521	249	1770			

Chi-square test was used to assess the association of variables; Φ: Effect size measure (Phi) and p value are reported; p: Level of significance.

## Data Availability

The data that support the findings of this study are available from the corresponding author upon reasonable request.

## References

[1] Belay WY, Getachew M, Tegegne BA, Teffera ZH, Dagne A, Zeleke TK, Abebe RB, Gedif AA, Fenta A, Yirdaw G, Tilahun A, Aschale Y (2024). Mechanism of antibacterial resistance, strategies and next-generation antimicrobials to contain antimicrobial resistance: A review. Front Pharmacol.

[2] Cassini A, Högberg LD, Plachouras D, Quattrocchi A, Hoxha A, Simonsen GS, Colomb-Cotinat M, Kretzschmar ME, Devleesschauwer B, Cecchini M, Ouakrim DA, Oliveira TC, Struelens MJ, Suetens C, Monnet DL (2019). Attributable deaths and disability-adjusted life-years caused by infections with antibiotic-resistant bacteria in the EU and the European Economic Area in 2015: A population-level modelling analysis. Lancet Infect Dis.

[3] Martine C, Sutherland S, Born K, Thompson W, Teoh L, Singhal S. Dental antimicrobial stewardship: A qualitative study of perspectives among Canadian dentistry sector leaders and experts in antimicrobial stewardship. JAC Antimicrob Resist. 2024;6(3):dlae082. 10.1093/jacamr/dlae082 PMC11109950 38779299

[4] Ahmadi H, Ebrahimi A, Ahmadi F (2021). Antibiotic therapy in dentistry. Int J Dent.

[5] Salam MA, Al-Amin MY, Salam MT, Pawar JS, Akhter N, Rabaan AA, Alqumber MAA (2023). Antimicrobial resistance: A growing serious threat for global public health. Healthcare (Basel).

[6] Buonavoglia A, Leone P, Solimando AG, Fasano R, Malerba E, Prete M, Corrente M, Prati C, Vacca A, Racanelli V (2021). Antibiotics or no antibiotics, that is the question: An update on efficient and effective use of antibiotics in dental practice. Antibiotics (Basel).

[7] Manciocchi E, Xhajanka E, D'Addazio G, Tafuri G, Santilli M, Rexhepi I, Caputi S, Sinjari B (2024). Antibiotic prescribing patterns among dentists in Italy and Albania: A comparative questionnaire analysis. Heliyon.

[8] Sukumar S, Martin F, Hughes T, Adler C (2020). Think before you prescribe: How dentistry contributes to antibiotic resistance. Aust Dent J.

[9] Teoh L, Stewart K, Marino R, McCullough M (2018). Antibiotic resistance and relevance to general dental practice in Australia. Aust Dent J.

[10] Al-Khatib A, AlMohammad RA (2022). Dentists' habits of antibiotic prescribing may be influenced by patient requests for prescriptions. Int J Dent.

[11] Amaral AL, Herrera D, Lund B, Andrade SA (2024). What is the science underpinning the prescription of antibiotics in dentistry?. Evid Based Dent.

[12] Albuquerque I, Melo P, Azevedo Á (2025). Antibiotic prescription habits of Portuguese oral healthcare professionals: An exploratory study. Rev Port Estomatol Med Dent Cir Maxilofac.

[13] Lockhart PB, Tampi MP, Abt E, Aminoshariae A, Durkin MJ, Fouad AF, Gopal P, Hatten BW, Kennedy E, Lang MS, Patton LL, Paumier T, Suda KJ, Pilcher L, Urquhart O, O'Brien KJ, Carrasco-Labra A. Evidence-based clinical practice guideline on antibiotic use for the urgent management of pulpal- and periapical-related dental pain and intraoral swelling: A report from the American Dental Association. J Am Dent Assoc. 2019;150(11):906-921.e12. 10.1016/j.adaj.2019.08.020 PMC8270006 31668170

[14] Teoh L, Cheung MC, Dashper S, James R, McCullough MJ (2021). Oral antibiotic for empirical management of acute dentoalveolar infections: A systematic review. Antibiotics (Basel).

[15] Nizami MZI, Yin IX, Niu JY, Yu OY, Chu CH (2025). Antibiotic resistance in dentistry: A review. Antibiotics (Basel).

[16] Duncan HF, El-Karim I (2025). Endodontic S3-level clinical practice guidelines: The European Society of Endodontology process and recommendations. Br Dent J.

[17] Sanz M, Herrera D, Kebschull M, Chapple I, Jepsen S, Berglundh T, Sculean A, Tonetti MS (2020). Treatment of stage I-III periodontitis-the EFP S3 level clinical practice guideline. J Clin Periodontol.

[18] Segura-Egea JJ, Gould K, Hakan Şen B, Jonasson P, Cotti E, Mazzoni A, Sunay H, Tjäderhane L, Dummer PMH (2018). European Society of Endodontology position statement: The use of antibiotics in endodontics. Int Endod J.

[19] American Academy of Pediatric Dentistry. Use of antibiotic therapy for pediatric dental patients. In: The Reference Manual of Pediatric Dentistry. Chicago: American Academy of Pediatric Dentistry; 2025. p. 559-63.

[20] Delgado V, Ajmone Marsan N, de Waha S, Bonaros N, Brida M, Burri H, Caselli S, Doenst T, Ederhy S, Erba PA, Foldager D, Fosbøl EL, Kovac J, Mestres CA, Miller OI, Miro JM, Pazdernik M, Pizzi MN, Quintana E, Rasmussen TB, Ristić AD, Rodés-Cabau J, Sionis A, Zühlke LJ, Borger MA (2023). 2023 ESC guidelines for the management of endocarditis. Eur Heart J.

[21] Abdullah FM, Hatim QY, Oraibi AI, Alsafar TH, Alsandook TA, Lutfi W, Al-Hussaniy HA (2024). Antimicrobial management of dental infections: Updated review. Medicine (Baltimore).

[22] Karapinar-Kazandag M, Tanalp J, Ersev H (2019). Effect of premedication on the success of inferior alveolar nerve block in patients with irreversible pulpitis: A systematic review of the literature. Biomed Res Int.

[23] Patra M, Gupta AK, Kumar D, Kumar B (2025). Antimicrobial resistance: A rising global threat to public health. Infect Drug Resist.

[24] Alhudaithi AS, Almutairi FJ, Almansour AS, Aljeadi AA, Kolarkodi SH (2025). Optimal duration of antibiotic therapy for space infections in the maxillofacial region: A systematic review. Clin Med Ther Rep.

[25] Rodríguez-Fernández A, Vázquez-Cancela O, Piñeiro-Lamas M, Herdeiro MT, Figueiras A, Zapata-Cachafeiro M (2023). Magnitude and determinants of inappropriate prescribing of antibiotics in dentistry: A nation-wide study. Antimicrob Resist Infect Control.

[26] Durkin MJ, Feng Q, Warren K, Lockhart PB, Thornhill MH, Munshi KD, Henderson RR, Hsueh K, Fraser VJ. Assessment of inappropriate antibiotic prescribing among a large cohort of general dentists in the United States. J Am Dent Assoc. 2018;149(5):372-381.e1. 10.1016/j.adaj.2017.11.034 PMC5995471 29703279

[27] Teoh L, Löffler C, Mun M, Agnihotry A, Kaur H, Born K, Thompson W (2025). A systematic review of dental antibiotic stewardship interventions. Community Dent Oral Epidemiol.

[28] Lim S, Awan D, Maling T (2022). A clinical audit and impact of interventions on antibiotic prescribing practices at a public dental primary care clinic. Arch Orofac Sci.

[29] Gelda A, Kadtane SS, Khichade PS, Singh V, Gupta D, Gupta G, Tripathy SS (2026). Assessment of antibiotic prescribing patterns among dentists and their impact on antimicrobial resistance in oral infections. J Pharm Bioallied Sci.

[30] Delbet-Dupas C, Devoize L, Depeyre A, Mulliez A, Barthélémy I, Pham Dang N (2019). Are routine microbiological samplings in acute dental infections justified? Our 10-year real-life experience. J Stomatol Oral Maxillofac Surg.

