# Supplementary material for: A retrospective analysis of antimicrobial prescriptions in dentistry: Understanding the patterns
Source: J Clin Exp Dent. 2026 Sep 15;18(10):e1231–7. doi: 10.4317/jced.64329 (PMC13739074; doi:10.4317/jced.64329)
Supplement: Supplementary file 1 [file jced-18-10-e1231-s001.pdf]

Supplemental Materials

Supplemental Table 1. Guidelines Used for Appropriateness Assessment of Clinical Conditions.

Clinical Condition	Source	Appropriate	Inappropriate	Indeterminate
Pulpal/periapical pain or swelling	ESE 2018	Prescription as an adjunct to local treatment when systemic involvement, spreading infection, progressive infection was documented.	Antibiotic prescribed for localized infection, dental pain, pulpitis, or periapical disease without systemic involvement.	Missing diagnosis, systemic signs, local treatment details, or antibiotic regimen.
Periodontal condition	EFP S3	Adjunctive therapy with mechanical treatment recorded.	Antibiotic use without documented periodontal indication.	Missing diagnosis, systemic signs, local treatment details, or antibiotic regimen.
Endocarditis prophylaxis	ESC 2023	High-risk cardiac condition and at-risk dental procedure documented; appropriate pre-operative prophylaxis prescribed.	Prophylaxis prescribed without documented indication, for a non-indicated procedure, or an incompatible regimen.	Missing diagnosis, procedure and antibiotic specifications.
Paediatrics	AAPD 2025	Documented facial swelling, systemic involvement, spreading infection, trauma-related indication, with age/weight-appropriate regimen.	Localized infection without systemic signs, non-bacterial condition, no clinical indication or inappropriate dosage/duration.	Missing diagnosis and antibiotic specifications.
